# Age dependent effects of early intervention in borderline personality disorder in adolescents

**DOI:** 10.1017/S0033291724000126

**Published:** 2024-07

**Authors:** Michael Kaess, Madelyn Thomson, Stefan Lerch, Julian Koenig, Gloria Fischer-Waldschmidt, Corinna Reichl, Marialuisa Cavelti

**Affiliations:** 1 University Hospital of Child and Adolescent Psychiatry and Psychotherapy, University of Bern, Bern, Switzerland; 2 Department of Child and Adolescent Psychiatry, Centre for Psychosocial Medicine, University of Heidelberg, Heidelberg, Germany; 3 Graduate School for Health Sciences, University of Bern, Bern, Switzerland; 4 Department of Child and Adolescent Psychiatry, University of Cologne, Faculty of Medicine and University Hospital Cologne, Psychosomatics and Psychotherapy, Cologne, Germany

**Keywords:** adolescence, age effects, borderline personality disorder, early intervention, natural course

## Abstract

**Background:**

Psychological treatments for young people with sub-threshold or full-syndrome borderline personality disorder (BPD) are found to be effective. However, little is known about the age at which adolescents benefit from early intervention. This study investigated whether age affects the effectiveness of early intervention for BPD.

**Methods:**

*N* = 626 participants (*M* age = 15 years, 82.7% female) were consecutively recruited from a specialized outpatient service for early intervention in BPD in adolescents aged 12- to 17-years old. DSM-IV BPD criteria were assessed at baseline, one-year (*n* = 339) and two-year (*n* = 279) follow-up.

**Results:**

Older adolescents presented with more BPD criteria (χ^2^_(1)_ = 58.23, *p* < 0.001) and showed a steeper decline of BPD criteria over the 2-year follow-up period compared with younger adolescents (χ^2^_(2)_ = 13.53, *p* = 0.001). In an attempt to disentangle effects of early intervention from the natural course of BPD, a parametrized regression model was used. An exponential decrease (*b* = 0.10, *p* < 0.001) in BPD criteria was found when starting therapy over the 2-year follow-up. This deviation from the natural course was impacted by age at therapy commencement (*b* = 0.06, *p* < 0.001), although significant across all ages: older adolescents showed a clear decrease in BPD criteria, and young adolescents a smaller decrease.

**Conclusions:**

Early intervention appears effective across adolescence, but manifests differently: preventing the normative increase of BPD pathology expected in younger adolescents, and significantly decreasing BPD pathology in older adolescents. The question as to whether developmentally adapted therapeutic interventions could lead to an even increased benefit for younger adolescents, should be explored in future studies.

## Introduction

Borderline personality disorder (BPD) is a severe mental disorder characterized by a pervasive pattern of affective instability, unstable interpersonal relationships and self-identity, as well as self-injurious and impulsive behavior. It is associated with a range of adverse outcomes including high psychiatric comorbidity, functional and psychosocial impairment, high risk of suicide, and overall premature mortality, and also incurs high burden for carers and families, as well as high treatment costs and resource use (Gunderson, Herpertz, Skodol, Torgersen, & Zanarini, 2018). Prevalence rates for BPD range from 1–3% in the general population, and up to 50% in inpatient settings (Sharp & Fonagy, 2015).

The natural course of symptoms of the disorder are broadly documented as follows: Precursors for BPD are already evident in childhood, and by early adolescence, BPD is clearly manifest and reliably distinguishable from the ‘storm and stress’ of normal adolescent development (Kaess, Brunner, & Chanen, 2014; Videler, Hutsebaut, Schulkens, Sobczak, & van Alphen, 2019). Symptoms tend to peak around mid- to late-adolescence (Stepp, Keenan, Hipwell, & Krueger, 2014), then largely attenuate over the adult years (Johnson et al., 2000), even though relapses in the further course, or an exacerbation in old age are possible (for a more detailed summary of the relevant literature regarding the course of BPD symptomology, please refer to eText 1 in online Supplementary Materials). Moreover, girls tend to mature faster than boys, particularly during the adolescent period (Lenroot & Giedd, 2010) which is also reflected in the development of psychopathology (Hayward & Sanborn, 2002) and personality development (Klimstra, Hale, Raaijmakers, Branje, & Meeus, 2009). Although possible that the symptomatic attenuation from adolescence to adulthood might be due to a natural decrease in impulsive behavior and attention seeking, and increases in self-control over the course of development, the presence of BPD pathology in adolescence already interrupts the healthy development of the person, and patients often present with high clinical distress and poor social and occupational functioning even if they only have sub-threshold BPD (Kaess, Fischer-Waldschmidt, Resch, & Koenig, 2017a; Thompson et al., 2018). Moreover, early onset BPD pathology (before 19 years of age) predicts long-term deficits in interpersonal, occupational, and general functioning (Winsper et al., 2015). Therefore, adolescence is a critical time window for early detection and intervention of BPD (Chanen & McCutcheon, 2013). Despite this, diagnosis and treatment of BPD in adolescents are often delayed due to clinician hesitation for fear of stigmatization associated with early diagnosis (Lustig, Koenig, Resch, & Kaess, 2021).

Early identification and intervention of BPD has recently become a global health priority in order to alter the course of the life trajectory for those with BPD (Chanen, Sharp, & Hoffman, 2017). There is now broad consensus that BPD is a valid and reliable diagnosis in adolescence (Wall, Leavitt, & Sharp, 2019), reflected in the absence of age restrictions in the current Diagnostic and Statistical Manual for Mental Disorders 5^th^ edition (DSM-5; American Psychiatric Association (APA)., 2013; Wall et al., 2019) and the upcoming International Classification of Diseases 11^th^ Revision (World Health Organization, 2018). Early intervention (both indicated prevention for sub-threshold BPD and treatment for first full presentation BPD) has been shown to be effective (Chanen et al., 2022; Fonagy et al., 2015). Several structured psychotherapeutic interventions have been developed for use with young people with BPD and many have demonstrated clinically significant improvements in both sub-threshold and full-threshold BPD, reducing BPD symptoms and improving psychosocial outcomes (Chanen, Nicol, Betts, & Thompson, 2020; Wall et al., 2019). The question arises as to whether early intervention is equally helpful for younger adolescents, as older ones, or whether there is too early an age for intervention (Thompson et al., 2018). To the best of our knowledge, there has not yet been an examination into the effect of age on early intervention for BPD in adolescence. This is surprising for two reasons: (i) age-effects of treatments have been demonstrated in other psychiatric disorders common in adolescents, such as depression (Curry et al., 2006), hinting that such effects might also be evident in BPD; (ii) the rapid developmental change (e.g. neurobiological and social), that occurs throughout adolescence and the consequent variations in neuropsychological abilities between younger and older adolescents, leads to the reasonable assumption that such developmental peculiarities might also affect the efficacy of early intervention for BPD (Curry et al., 2006; Sharp, Vanwoerden, & Wall, 2018). Further, given the longstanding controversy surrounding the BPD diagnosis at young ages, more data on the effects of early diagnosis of BPD on treatment outcomes is warranted.

To address this gap, the aim of the current study was twofold: First, to explore whether age at intervention affects the course of BPD over two years, in the context of a clinical cohort study on adolescents; and second, to disentangle ‘early intervention’ effects from the natural course of the disorder. While we acknowledge that a randomized controlled trial would be the ideal method to examine this question, ethical limitations (i.e. placing help-seeking adolescents with borderline features on a long waitlist to investigate their normative course of BPD) do not permit this. Therefore, we have developed a way to explore this question using a novel statistical approach involving parametrization. Further, given the sex differences in maturation, we sought to explore the natural course of BPD symptomology, as well as the impact of intervention, for both sexes.

## Method

### Participants and procedures

Participants were consecutively recruited from a specialized outpatient clinic for early intervention for adolescents with emerging BPD symptoms or first presentation BPD at the Clinic of Child and Adolescent Psychiatry, University Hospital Heidelberg, Germany (AtR!Sk; *Ambulanz für Risikoverhalten und Selbstschädigung*). The service provides low-threshold initial contact, detailed and comprehensive diagnostic assessment of BPD features and evidence-based therapeutic intervention (Kaess, Ghinea, Fischer-Waldschmidt, & Resch, 2017b). In line with the stepped care approach, participants were offered tailored intervention depending on symptom severity and treatment response (Kaess et al., 2017b). This included initial short-term cognitive-behavioral therapy (brief CBT; Kaess et al., 2020; Rockstroh et al., 2023), which was followed by Dialectical Behavioral Therapy for Adolescents (DBT-A; Buerger et al., 2019) for those with persistent symptoms, along with psychiatric management and specialist crisis involvement (e.g. outpatient crisis interventions or time-limited admission to the acute ward) where necessary. However, this consecutively recruited clinical cohort also included patients who diverged from this standard procedure by not engaging in proposed interventions, dropping out from treatment early, or needing inpatient treatment. Inpatient treatment comprised of a less standardized but multimodal, interdisciplinary treatment that was conducted in accordance with the DBT-A principles. In addition to this, both DBT-A informed individual psychotherapy and skills group were conducted weekly.

The AtR!Sk cohort study was conducted in accordance with the Declaration of Helsinki (World Medical Association, 2013), and approved by the ethics committee of the Medical Faculty of the University of Heidelberg, Germany (ID S-449/2013). Inclusion criteria were: 12–17 years of age and participation in the AtR!Sk diagnostic phase. Exclusion criteria were: insufficient German language skills; acute psychotic disorder and/or intention to commit suicide or intention to harm others, requiring immediate inpatient admission; impairment of intellectual functioning; and diagnosis of bipolar disorder, schizophrenia, or schizoaffective disorder. Written informed consent (or assent, respectively) was obtained from all participants, and also from a parent or legal guardian for those under the age of 16 years. Initial baseline assessments were part of the usual diagnostic assessment procedure of the AtR!Sk clinic. Further assessments were conducted at follow-up 1 (one year after baseline) and follow-up 2 (two years after baseline), with diagnostic assessments conducted by clinical psychologists. Participants were reimbursed 20 Euros for each follow-up interview.

### Measures

Demographic information was collected using a standardized set of interview questions assessing age, sex, school type, family, and living situation. Our primary outcome was the number of DSM-based BPD criteria, as assessed by the German version of the Structured Clinical Interview for DSM-IV Axis II Personality Disorders (SCID-II; First, Spitzer, Gibbon, Williams, and Benjamin, 1994; Fydrich, Renneberg, Schmitz, and Wittchen, 1997). It reflects the clinical diagnostic criteria for BPD in the DSM-IV that have remained unchanged in version 5. It is one of the most widely used semi-structured interviews for the assessment of borderline pathology, across clinical and research settings for both adults and adolescents (Fonagy et al., 2015). The SCID-II BPD module takes about 10–15 min to administer, has been translated into multiple languages, has shown adequate inter-rater reliability and internal consistency, and, as the categorical diagnosis of BPD requires five of nine criteria to be present in the individual, a change in score could not only potentially reflect differences in being diagnosed/not diagnosed with the disorder, but also severity, with sub-threshold scores being important indicators of personality pathology (Maffei et al., 1997; Thompson et al., 2018). The Mini-International Neuropsychiatric Interview for Children and Adolescents (M.I.N.I.-KID; Sheehan et al., 2010) was used to assess psychiatric disorders according to the ICD-10 (International classification of diseases and related health problems (World Health Organization, 1992). The Self-Injurious Thoughts and Behaviors Interview (German version; SITBI-G; Fischer et al., 2014) was used to measure the occurrence, frequency and characteristics of suicidal ideation, plans and attempts, as well as thoughts about non-suicidal self-injury (NSSI), and manifest NSSI. The presence of Suicidal Behavior Disorder and NSSI disorder were defined according to DSM-5 (American Psychiatric Association (APA)., 2013). The German version of this interview has demonstrated good psychometric properties (Fischer et al., 2014).

### Statistical analyses

First, to examine the effect of age on the course of BPD over two years, a mixed-effects binomial linear regression analysis with nine trials and a logit-link function was used. Participants with missing data were included in the analyses (i.e. usage of available data), with their observed values used to determine the longitudinal trajectory through the use of random effects. In the current analyses, time point (discrete fixed-effect factor with three levels: baseline (0), 1-year follow-up (1), and 2-year follow-up (2)), age (years), time point *x* age interaction, and sex (m/f) served as the fixed factors, and observations were grouped by subjects, allowing for a random intercept and random slope for time. Time was treated as categorical for the fixed effect and continuous for the random slope. Number of fulfilled DSM-IV BPD criteria (between zero and nine criteria) was the dependent variable. Contrasts were undertaken using the Wald tests.

Second, to disentangle therapy effects from the natural course of BPD over time, a series of nonlinear mixed-effect regression analyses with parameterization were conducted, based on the same data as in the first analysis (baseline, 1-year follow-up, and 2-year follow-up). The parameterized functions were chosen to represent (i) the natural course of BPD symptoms across adolescence, (ii) the therapy effect (i.e. a deviation from the natural course as soon as therapy starts), and (iii) a sex effect on both the natural course and the therapy effect. A two-step approach was applied. First, variations of estimated models were constructed and tested. These models were informed by the literature regarding the natural course of BPD symptoms (e.g. linear increase, plateau, etc.), and the suitability of each model was constructed and tested using baseline data only, with the best fitting model determined using goodness-of-fit indices. Second, the model was extended by the inclusion of follow-up data allowing to estimate a potential deviation from the natural course after therapy commencement (i.e. the therapy effect), and, again, models were compared to determine the best fitting option. For further details regarding the model construction and comparison, please see eText 2 and accompanying eFigures and eTables in the online Supplementary Materials.

The final, best-fitting parameterized model used to describe the number of fulfilled BPD criteria is described in more detail below, and presented in the results.
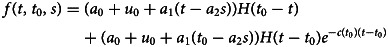


The intercept *a_0_* and the slope *a_1_* captures the linear increase in BPD criteria reflecting the natural course. The natural course potentially differs (*a*_2_) between sexes (*s*), (0 = female, 1 = male). As soon as therapy starts, the course is described by an exponential decay rate (*c*(*t*_0_)), reflecting a potential decrease in BPD criteria as the result of therapy (i.e. therapy effect). The step function *H* controls if the therapy is active or not:
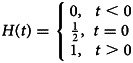


The decay rate *c* (i.e. the therapy effect) depends on age:
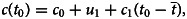
where *c*_0_ is the decay constant for a subject starting the therapy at 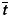 = 15 years (mean age in our sample) and *c*_1_ determines the change in the decay rate, when not starting therapy at 15 years of age (*c*_0_). Both 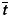 and *t*_0_ are values on the continuous time variable *t*, where *t* = 0 corresponds to the subject's birth (i.e. age of zero), *t*_0_ denotes the age at commencement of therapy, and 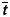 allows for the interpretation of the decay rate *c*_0_ at the age of 15 years. That is, in the data, *t* and *t*_0_ are observed variables, and 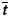 is an arbitrary constant to aid interpretation of the effect *c*_0_.

Random effect *u*_0_ captures individual differences in the number of fulfilled BPD criteria in the natural course before the therapy started, meaning a subject with positive *u*_0_ presents with more fulfilled BPD criteria at all timepoints than a subject with smaller *u*_0_. Random effect *u*_1_ allows for individual change rates of BPD criteria as soon as therapy starts, with a positive *u*_1_ indicating a steeper decrease. In the current analysis, we used age at baseline as *t*_0_ and the time difference between the timepoints to set up the time variable *t*. *a*_0_, *a*_1_, *a*_2_, *c*_0_, *c*_1_ were used as fixed effects, *u*_0_, *u*_1_ as random effects.

Finally, in order to investigate the potential confounding effect of treatment dose on the association between age and the therapy effect (i.e. decay rate *c*), we re-ran the analyses with treatment dose as an additional predictor. Detailed information on this sensitivity analysis is provided in the eText 3 of the online Supplementary Materials. STATA version 17 (Stata Statistical Software:, 2019) was used for all analyses. The significance level was set at *α* = 0.05.

## Results

### Participants

Of 782 patients, 678 agreed to participate in the longitudinal AtR!Sk cohort study (86.7%). Among those, 626 completed the baseline assessment before the recruitment stop on 31/12/2019. Seven participants were excluded from the analyses due to missing age or sex data, or due to incorrect age at baseline (included age range 12–17 years at baseline). Among the *n* = 619 remaining participants, *n* = 339 (51%) were assessed at one-year follow-up. Additionally, *n* = 279 participants (74% of the sample at one-year follow-up) were assessed at second follow-up. The mean age of participants at baseline was 15.02 years (*SD* = 1.42; range = 12–17 years) and the majority of the sample were female (*n* = 510; 82.7%). For further participant information, see Table 1.

**Table 1.** Sociodemographic, clinical and treatment variables of the sample

	Baseline (*n* = 619)	Follow-up 1 (*n* = 339)	Follow-up 2 (*n* = 279)
*Sociodemographic variables*	*n* (*%*)	*n* (*%*)	*n* (*%*)
School type^a^			
Hauptschule	68 (11.0)		
Realschule	215 (34.7)		
Gymnasium	218 (35.2)		
Other	115 (18.6)		
Living with:			
Both parents	260 (42.0)		
Mother only	185 (29.9)		
Father only	29 (4.7)		
Other (i.e. step-parents, etc.)	82 (13.2)		
*Clinical variables*			
BPD diagnosis	209 (33.8)		
Number of fulfilled BPD criteria (M ± 1SD)	3.53 ± 2.26		
NSSI disorder^b^	470 (75.9)		
Number of NSSI in the past year (M ± 1SD)	59.31 ± 76.10		
Suicidal behavior disorder^c^	227 (36.7)		
Suicide attempts in the past year (M ± 1SD)	1.54 ± 7.86		
Number of psychiatric diagnoses (M ± 1SD)	2.49 ± 1.62		
Co-occurring mental disorders (ICD-10 categories)			
F0 (mental disorders due to known physiological conditions, e.g. dementia)	0		
F1 (substance related disorders, e.g. drugs or alcohol)	143 (23.1)		
F2 (psychotic disorders)	0		
F3 (affective disorders)	382 (61.7)		
F4 (anxiety, dissociative, stress-related, somatoform, and other nonpsychotic mental disorders)	238 (38.4)		
F5 (behavioral syndromes associated with physiological disturbances and physical factors, e.g. eating disorders, sleep disorders, sexual dysfunction)	78 (12.6)		
F6 (personality disorders) other than BPD	278 (44.9)		
F7 (intellectual disabilities)	0		
F8 (pervasive and specific developmental disorders, e.g. disorders of speech and language, scholastic skills)	3 (0.48)		
F9 (Behavioral and emotional disorders with onset usually occurring in childhood and adolescence, e.g. ADHD, conduct disorder)	203 (32.8)		
*Treatment variables*^d^			
Psychotropic medication (yes)	59 (9.5)	67 (19.8)	63 (22.6)
Number of participants with inpatient treatment in the past year		129 (38.1)	53 (19.0)
Of those, inpatient days in the past year (M ± 1SD)		64.22 ± 59.54	71.78 ± 78.10
Number of participants with outpatient treatment in the past year		244 (72.0)	152 (54.5)
Of those, outpatient sessions attended in the past year (M ± 1SD)		23.96 ± 18.37	34.99 ± 27.20

*Notes*: M = mean, SD = standard deviation.

a Hauptschule, nine years of elementary school; Realschule, six years of school after four years of elementary school, terminating with a secondary school level-I certificate; Gymnasium = eight years of school after four years of elementary school, terminating with the general qualification for university entrance.

b Non-suicidal self-injury disorder, as determined by DSM-5.

c Suicidal behavior disorder, as determined by DSM-5.

d There is an overlap in treatment (outpatient/inpatient) for some participants.

To check for a systematic loss of participants, participants with baseline data only were compared to participants with at least one follow-up assessment with respect to sociodemographic and clinical characteristics. There were no relevant group differences, except that participants with baseline data only (i.e. no follow-up data, dropouts) were significantly more likely to have an ICD-10 F9 diagnosis (‘Behavioral and emotional disorders with onset usually occurring in childhood and adolescence’, e.g. attention-deficit hyperactivity disorders, conduct disorders), than those with at least one follow-up assessment. Detailed information on the dropout analysis is provided in eText 4 of the online Supplementary Materials.

### The effect of age on the course of BPD

**Figure 1.**
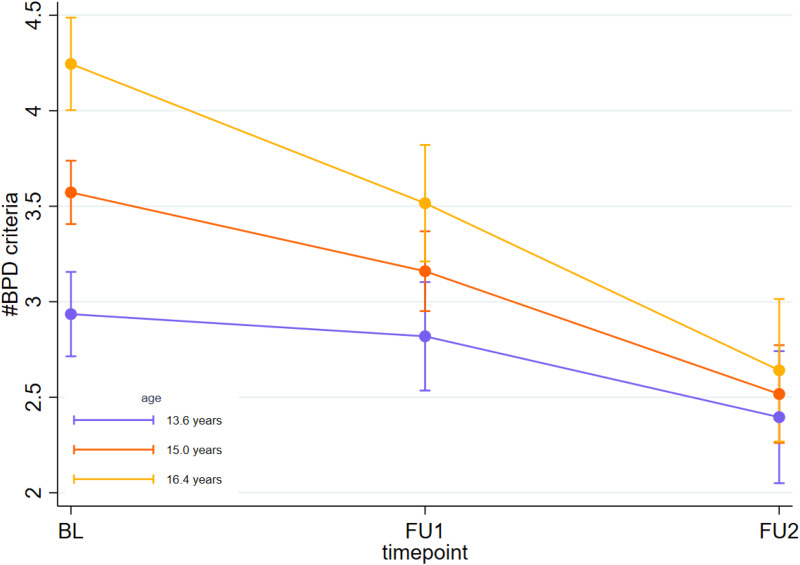
The effect of age on the course of BPD over two years. *Note:* Marginal predicted mean number of fulfilled BPD criteria for youth at different ages (15 years = mean, 13.6/16.4 years = mean -/+1 standard deviation of the sample), according to the fixed portion of the binomial linear regression. Error bars denote 95% confidence intervals.

The overall regression model predicting the number of BPD criteria by timepoint, age, the timepoint *x* age interaction, and sex was significant (χ^2^_(6)_ = 161.13, *p* < 0.001). A significant main effect for timepoint (χ^2^_(2)_ = 8.87, *p* = 0.012) was found, with the number of fulfilled BPD criteria decreasing from baseline to the 2-year follow-up. Further, a significant main effect of age at baseline (χ^2^_(1)_ = 58.23, *p* < 0.001) was found, with older adolescents having higher numbers of fulfilled BPD criteria than younger ones at all timepoints. Additionally, a significant sex effect was found (χ^2^_(1)_ = 70.44, *p* < 0.001), where males had on average 1.0 ( + /- 1SD = 0.1) criterion fewer fulfilled than females at all timepoints. Finally, analyses yielded a significant timepoint *x* age interaction effect (χ^2^_(2)_ = 13.53, *p* = 0.001). Although age was measured as a continuous variable, for illustrative purposes, three age values were selected (in the following referred to as ‘age groups’), namely mean sample age (15 years), plus/minus one standard deviation (13.6 years/16.4 years). As illustrated in Fig. 1, older adolescents showed the steepest decline in number of fulfilled BPD criteria across timepoints (significant), followed by middle adolescents (still significant), and younger adolescents (non-significant). Refer to Table 2 for changes in the mean number of BPD criteria fulfilled between timepoints for the three selected age groups.

**Table 2.** Changes in the number of fulfilled BPD criteria as a function of timepoints by separate age groups (*N* = 619)

	Age group (based on age at baseline)		
	M-1SD (13.6 years)	M (15 years)	M + 1SD (16.4 years)
Mean number of fulfilled BPD criteria (standard error)			
Baseline	2.90 (0.11)	3.55 (0.09)	4.24 (0.13)
Follow-up 1	2.81 (0.15)	3.15 (0.11)	3.51 (0.16)
Follow-up 2	2.51 (0.17)	2.63 (0.12)	2.75 (0.18)
Change in mean number of fulfilled BPD criteria between timepoints (standard error)			
Follow-up 1 v. Baseline	−0.09 (0.14), *p* = 0.518	−0.40 (0.11), *p* < 0.001	−0.73 (0.15), *p* < 0.001
Follow-up 2 v. Follow-up 1	−0.29 (0.16), *p* = 0.072	−0.52 (0.12), *p* < 0.001	−0.76 (0.17), *p* < 0.001
Follow-up 2 v. Baseline	−0.39 (0.18) *p* = 0.028	−0.92 (0.13) *p* < 0.001	−1.49 (0.19) *p* = 0.001

*Note*: Age was measured as a continuous variable; however, three different age groups were selected for illustrative purposes.

### Disentangling therapy effects from the natural course of BPD

**Figure 2.**
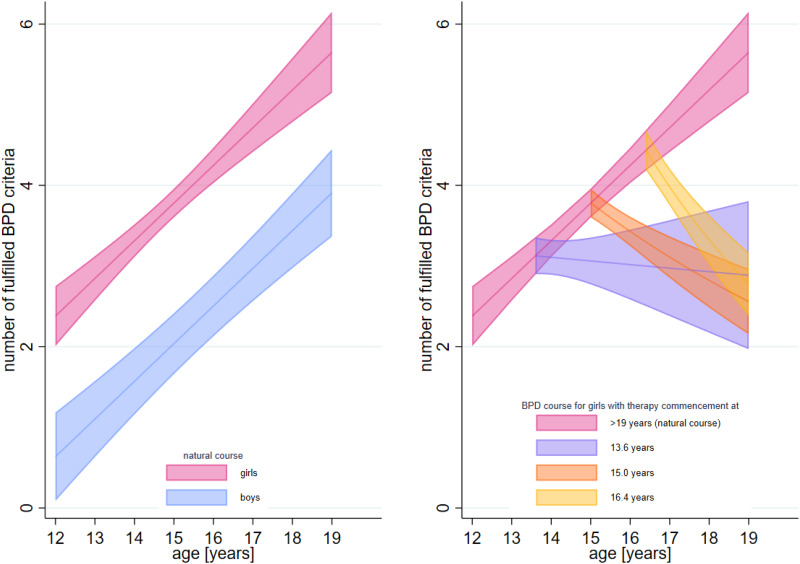
Effect of age at intervention measured against the natural course of BPD. *Note.* Interpolated marginal predicted mean number of fulfilled BPD criteria for an individual at different ages according to the fixed effects of the nonlinear mixed-effects regression. The left plot illustrates the natural courses when no therapy takes place for girls (pink) and boys (blue). The right plot illustrates the deviation of the natural course for a girl (given that there was no sex effect found for the follow-up data (i.e. therapy) inclusion), starting therapy at different ages (same age groups as in Fig. 1; orange (mean sample age), purple (mean sample age – 1 standard deviation) and yellow (mean sample age + 1 standard deviation)). Shadowed regions indicate the 95% confidence interval.

The final, best-fitting parameterized regression model predicting the number of BPD criteria was significant (χ^2^_(6)_ = 327.82, *p* < 0.001). The estimates of the parametrized model are displayed in Table 3. The natural course showed a linear increase of 0.47 BPD criteria per year (*a_1_*). The natural increase was delayed for boys by 3.73 years (95% CI = 2.58–4.88) compared to girls (*a_2_*). The decrease in BPD criteria starting with therapy (*c*_0_; i.e. therapy effect at the age of 15) was significant. As can be seen in Fig. 2, with the beginning of therapy, the course of BPD (orange, purple, and yellow lines for the selected age groups) deviated from the assumed natural course (pink curve). *c*_1_ reflecting different decrease rates in BPD criteria according to the age at therapy commencement was significant, indicating that the therapy effect was impacted by the age at intervention: the later age that a person starts therapy, the greater the deviation from the natural course (i.e. therapy effect). Consequently, older adolescents (i.e. 16.4 years age group in Fig. 2) demonstrated a clear decrease in BPD criteria, and young adolescents (i.e. 13.6 years age group in Fig. 2) a smaller decrease. However, it should be noted that for very young adolescents (i.e. 12 years) they showed an increase in criteria rather than a decrease, although a smaller increase than would have been expected for the natural course. Regardless, the deviation from the natural course in BPD criteria through therapy was significant for the entire age range.

**Table 3.** Estimates and errors for non-linear mixed-effects regression (*N* = 619)

BPD parametrization	Coefficient	Standard error	*z*	*p*	95% CIs	
					Lower	Upper
Natural course, intercept (*α_0_*)	−3.23	0.87	−3.72	<0.001	−4.93	−1.53
Natural course, slope (*α_1_*)	0.47	0.06	8.05	<0.001	0.35	0.58
Time shift between boys and girls (*a*_2_)	3.73	0.59	6.36	<0.001	2.58	4.88
Decrease resulting from therapy (*c_0_*)	0.10	0.02	4.66	<0.001	0.06	0.14
Age dependency of therapy effect (*c_1_*)	0.06	0.02	3.86	<0.001	0.03	0.09
Variance of random effect (*u_0_*)	2.43	0.24			2.00	2.95
Variance of random effect (*u_1_*)	0.04	0.01			0.03	0.07

*Note*: Main therapy effect (*c_0_*) refers to an age of 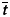 set at 15 years (mean age).

The results remained unchanged when treatment dose was considered as a covariate (see eText 3 of the online Supplementary Materials). This indicates that the effect of age on the therapy effect cannot be explained by differences in treatment dose.

## Discussion

To our knowledge, this is the first study to examine the effect of age in evidence-based early intervention of BPD in adolescents. Four main findings emerge from the analyses. First, older adolescents (around 16.5 years) presented with more BPD criteria at commencement of therapy compared to younger adolescents (around 13.5 years). Second, girls presented with more BPD criteria than boys. When examined in regards to the natural course, the sex effect was explained by the natural increase in criteria being delayed for boys. Third, we found a significant therapy effect across all age ranges in adolescence defined as a deviation from the natural course when starting therapy, and this was not dependent on sex. Finally, the therapy effect was dependent on age at intervention. Specifically, the older the adolescent at age of therapy commencement, the greater the decline in BPD criteria over the two years. That is, it appeared that intervention prevented the normatively expected increase of borderline pathology expected in younger patients, and led to a reduction in borderline pathology in older patients. Therefore, we conclude that early intervention had beneficial effects across adolescence. For younger patients, a stable or a smaller increase of BPD pathology than what would be normatively expected appears to be a gain of treatment, and for older adolescents, a reduction in symptoms is a clear benefit from early intervention. These results support broader research concluding that early intervention is beneficial for young people with BPD pathology, and extends current knowledge to the proof of a potential benefit of even earlier intervention (Chanen et al., 2020; Fonagy et al., 2015; Wall et al., 2019). Aligning with clinical staging (Hutsebaut, Videler, Verheul, & Van Alphen, 2019), our results suggest that indicated prevention at early ages when BPD symptoms often do not reach the threshold for full BPD diagnosis, may prevent young individuals from developing the disorder at all.

### Clinical implications

Existing evidence encourages early detection and treatment of BPD pathology in adolescence, and our findings extend on this by demonstrating that this is justified even in younger adolescents. Clinicians can be mindful that for middle and older adolescents, early intervention may help reduce symptoms, and for younger adolescents it may act as a buffer, preventing further development of BPD symptoms. In other words, clinicians might consider stability in BPD pathology – rather than a reduction – in younger persons, a goal of treatment.

### Strengths and limitations

Clear strengths of the current study are the large sample size and its longitudinal nature. Additionally, the inclusion of young (from 12 years of age) through to older adolescents enables examination of therapy effects across the course of the adolescent period. Additionally, the use of gold standard structured diagnostic assessments, and that they were conducted by well-trained psychologists provides strong validity to the data. Finally, we modeled several variations of both the natural course with baseline data, as well as the therapy effect by extended models including follow-up data, and determined the best fit for the sample, ensuring that the final model is indeed the most appropriate selection for the data.

Some limitations should also be considered. First, as with most other studies on BPD, the current study had a skewed sex ratio towards female. However, sex was included in the analyses with clear sex differences found – males endorsed on average, one fewer BPD criterion than females. Furthermore, boys showed a delayed maturation (of approximately 3.7 years) compared to girls when represented on the parameterized model comprising the natural course of BPD symptoms (although no sex effect was found for the therapy component of the model). Second, the parameterized model for the natural course allowed for negative numbers of BPD criteria, as well as more than nine BPD criteria (both not possible), due to the limited age range in our data, and therefore, extrapolation is not possible. Therefore, results should be interpreted with caution for ages below 11 and above 19 years (i.e. 17 years plus the two years follow-up). It should also be noted that the model in our study is estimated with our particular sample who commenced therapy soon after baseline assessment, and thus, we assume that the natural course is well described by the participants presenting to the service. However, it is unknown as to how this might change based on other samples including different age ranges at time of therapy presentation, or even non-help-seeking samples. Third, the current study was an uncontrolled cohort study, and therefore attributing the observed symptomatic improvement to treatment with certainty, is not possible. Although it is also worth reiterating that a ‘no treatment’ group in help-seeking patients with BPD is considered unethical. Fourth, dropout analyses revealed that those having only baseline data (i.e. having not completed a follow-up) were more likely to have an ICD-10 F9 diagnosis. Given that F9 diagnoses include those with Attention-Deficit/Hyperactivity Disorder, this result is understandable, in that such individuals might have difficulties in sustaining attention or interest for further follow-up assessments that are optional, rather than part of initial clinical diagnostics. Regardless, this might reflect selection bias and our results may not be generalizable to this specific group. Furthermore, while the focus on symptomatic change in our study has important clinical implications, we did not include other relevant outcomes such as recovery, functioning, or quality of life into the model, which should be examined in future research.

### Future directions

The current study supports indicated prevention and early intervention for adolescents with emerging BPD at all ages. However, it raises the question as to whether developmentally adapted therapeutic interventions could lead to increased benefit for younger adolescents who did not show the normatively expected rate of increase in BPD symptoms, but neither showed a decrease in BPD symptoms. Considering the vast differences in cognitive and emotional abilities across adolescence, developmental peculiarities must be considered when designing interventions for BPD in adolescents (Sharp et al., 2018). For example, for younger adolescents there may be more emphasis on family involvement, whereas for middle and older adolescents, therapy focused on mentalization and formation of self-identity coinciding with expanding cognitive and social abilities, might be more useful. Thus, effects of early intervention seen across different age groups should be further examined to determine the mechanisms for change within each age group (young, middle, and older adolescents).

## Conclusion

Early intervention appears effective from early to late adolescence, with its effectiveness manifesting in differing ways for younger and older adolescents: younger ones gaining a stability of pathology rather than increasing, and older ones experiencing a steady reduction of symptoms over time. Our results support the early intervention of BPD in young people, in that it appears that there may not be an age that is considered ‘too early’ for intervention, with benefits seen across the course of adolescence. Therefore, clinicians should not be hesitant to accurately diagnose and treat BPD symptomology in a timely manner (that is, where clinical evidence indicates its presence in adolescence). Overall, results emphasize the benefits of diagnosing and treating this frequently debilitating disorder, in that it may alter the trajectory for many patients, regardless of age of intervention.

## Supporting information

Kaess et al. supplementary material Kaess et al. supplementary material

## Data Availability

Due to the nature of this research project, participants did not provide consent for their data to be shared publicly, so supporting data is not publicly available and therefore, not open access. However, anonymized data can be made available upon request from the corresponding author.

## Supplementary material

The supplementary material for this article can be found at https://doi.org/10.1017/S0033291724000126.
