# Supplementary material for: Age dependent effects of early intervention in borderline personality disorder in adolescents
Source: Psychol Med. 2024 Feb 12;54(9):2033–41. doi: 10.1017/S0033291724000126 (PMC11413336; doi:10.1017/S0033291724000126)
Supplement: Kaess et al. supplementary material [file S0033291724000126sup001.docx]

**Supplementary Material for**

***“Age dependent effects of early intervention in borderline personality disorder in adolescents”***

Michael Kaess, Madelyn Thomson, Stefan Lerch, Julian Koenig, Gloria Fischer-Waldschmidt, Corinna Reichl, Marialuisa Cavelti

Content: 4 eTexts, 3 eFigure, 3 eTables

- eText 1: A brief summary of the literature on the course of Borderline Personality Disorder symptoms
- eText 2: Building the parametrized model and determining the best fit (includes the following: eFigure 1, eTable 1, eFigure 2, eTable 2, eFigure 3, eTable 3)
- eText 3: Effect of age on treatment intensity (dose): sensitivity analyses
- eText 4: Check for systematic loss of participants

**eText1: A brief summary of the literature on the natural course of Borderline Personality Disorder symptoms**

The natural course of BPD symptoms has been documented in the literature over the past two decades mostly comprising community studies. For example, regarding prevalence, a cross-sectional study of community residents in Germany (*N*=2,488) revealed that prevalence of BPD (as assessed by standardised clinical interviews) has a peak in adolescence (5.4%), followed by a sharp decline into young adulthood (0.9%), and further prevalence reductions into middle (0.3%) and old (0%) age (Arens et al., 2013). Longitudinal data also support this peak in adolescence. In the Pittsburg Girls Study, a prospective community study of girls (*N*=2,450) in the United States, which used parent and teacher reports for the children at the first wave (when they were 5-8 years old), and child reports thereafter, they found a peak of BPD symptoms by age 15, declining through age 18, then levelling off between the ages of 18 and 19 years (Stepp et al., 2014). The Children in the Community Study (Johnson et al., 2000) which assessed 816 youths in a representative community sample, found the highest mean rates of borderline traits in early to mid-adolescence which then linearly declined into adulthood. Finally, using a large community sample of twins aged assessed over a 10-year period (from age 14-24 years) as part of the Minnesota Twin Family Study, BPD traits were found to significantly decline from adolescence to adulthood (Bornovalova et al., 2009).

In contrast, a recent meta-analysis found a peak of BPD symptoms at the age of 29.4 years, rather than through mid-late adolescence (Aleva et al., 2022). However, cross-sectional data only was used, as longitudinal data that was collected were too limited to conduct meaningful testing. Authors also noted that the interpretation of the identified peak is uncertain, and that statistical limitations might account for some of the results. Specifically, the results of the overall model were suggested to be a possible statistical artefact, given that subsequent distinctive models emerged when the context of a sample (BPD, community, and patient) was considered. Therefore, due to the combination of different sample contexts included in the analyses, this may have resulted in insufficient sensitivity to the factor of context. That is, specifically, as there was a disproportionately high amount of studies included with a mean age of 30 years, therefore, by definition, having higher mean-levels of BPD features. Additionally, the model could not be replicated when including only samples with limited age-variability (i.e., samples falling below the cut-off value of standard deviation of age < 5 years).

It should also be noted that variability and waxing and waning of remission, recovery and relapse of BPD over the lifespan are not uncommon (Álvarez-Tomás et al., 2019; Temes & Zanarini, 2018; Videler et al., 2019). Indeed, categorical personality diagnoses (including BPD) have been found to be more unstable than previously assumed, in both adults and adolescents (d’Huart et al., 2023). Moreover, despite a trend towards attenuation over time, ‘recovery’ should not necessarily be assumed, as deleterious long-term effects persist, including symptomatic shifts, and might even be exacerbated in old age (D’Agostino et al., 2022; Videler et al., 2019). A recent meta-analysis found that while symptomatic amelioration of BPD was characteristic of the disorder, it was also accompanied only by slight improvements in functional improvements (Álvarez-Tomás et al., 2019). This is particularly the case regarding impairments in social and vocational functioning (Alvarez-Tomás et al., 2017) that often persist even decades on when BPD features are no longer clinically evident (Gunderson et al., 2011; Soloff & Chiappetta, 2019; Zanarini et al., 2010, 2018).

Overall, some details of the natural course are still unclear and require more detailed longitudinal studies covering the lifespan of BPD symptomology. Particularly, it should be acknowledged that there is a paucity of longitudinal studies examining the potential fluctuations in BPD symptomology that might be expected in the adolescent period, which is marked by rapid neurobiological, physical, psychological and social changes. Regardless, the broader literature to date using large samples supports the general course consisting of a peak in mid- to late-adolescence, then a natural tapering off over the early adult years, and recent reviews have largely supported this conclusion (Videler et al., 2019; Winsper, 2021), with adolescence also considered to be a sensitive period for the development of personality disorders (Sharp et al., 2018).

**eText 2: Building the parametrized model and determining the best fit**In order to construct a parametrized model that (i) the natural course of BPD symptoms across adolescence (ii) the therapy effect (i.e., a deviation from the natural course as soon as therapy starts), and (iii) a sex effect on both the natural course and the therapy effect, a two-step approach was applied. Herein, we outline the discrete steps taken to develop the final model:

1. First, we modelled baseline data only in order to estimate the *natural course of BPD symptoms*, using different variations of parametrizations (broadly informed by the natural course of symptoms as described in the literature). In eFigure 1, variations of possible models are presented with simulated data of BPD criteria for illustrative purposes. Refer to eTable 1 for an outline of the description and corresponding mathematical formulas for each model.

**eFigure 1:** *Variations of parametrized models to estimate the natural course of BPD symptoms using simulated data for illustration purposes*

*Notes*: Illustration of the models in eTable 1 on simulated data. y-axis: number of fulfilled BPD criteria. Simulated data are illustrated as shaded points. In the top row the model does not differ between sex. In the bottom row the model allows for a time shift between girls (red) and boys (blue). Data is modelled by a linear slope (M1), by an increase and later decrease (M2), by an increase until it levels out at some point and decreases again (M3), an exponential decay that interacts with a linear increase (M4), and an increase that levels out (M5).


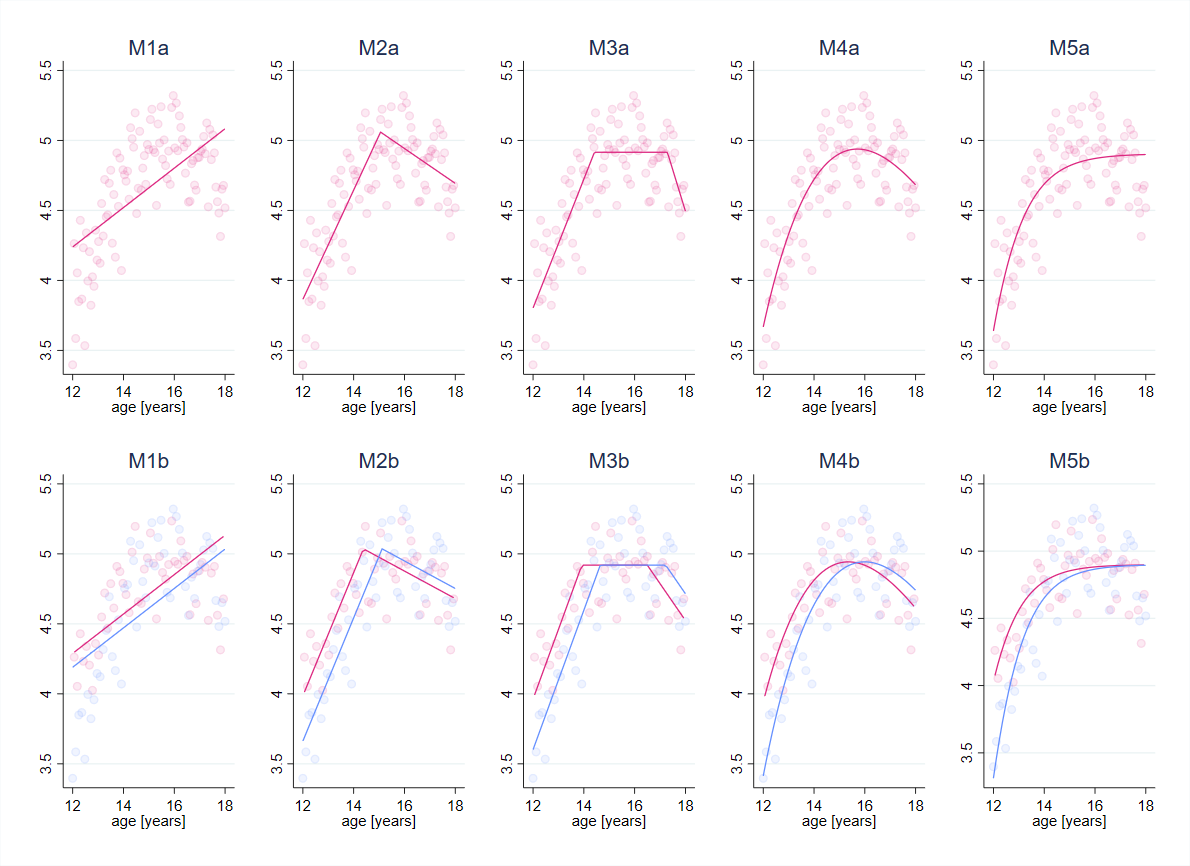


**eTable 1:** *Outline of each model as depicted in eFigure 1*

| Model | Description | Mathematical formula |
| --- | --- | --- |
| M1: Linear | An increase of BPD criteria with a constant rate over time. | M1b: $f\left( t,s \right)=\left( a_{0}+a_{1}(t-a_{2}s) \right)$  M1a: M1b setting $a_{2}=0$ |
| M2: Breakpoint | An increase of BPD criteria with a distinct breakpoint or change point where the nature of the relationship between variables (i.e., BPD over time) changes. | M2b: $f\left( t,s \right)=\left\{ \begin{matrix} a_{0}+a_{1}(t-a_{2}s) & if t\leq a_{3}+a_{2}s \\ a_{4}+a_{5}(t-a_{2}s) & if t>a_{3}+a_{2}s \end{matrix} \right.$  $a_{4}=a_{0}+a_{1}a_{3}-a_{5}a_{3}$  M2a: M2b setting $a_{2}=0$ |
| M3: Plateau | A linear increase of BPD criteria with a levelling off at a particular point over a time interval (i.e., the plateau) before decreasing again. | M3b: $f\left( t,s \right)=\left\{ \begin{matrix} a_{0}+a_{1}(t-a_{2}s) & if t\leq a_{3}+a_{4}s \\ a_{5} & if a_{3}+a_{4}s<t<a_{6}+a_{4}s \\ a_{7}+a_{8}(t-a_{2}s) & if t\geq a_{6}+a_{4}s \end{matrix} \right.$  $a_{5}=a_{0}+a_{1}a_{3}$  $a_{7}=a_{5}-a_{8}a_{6}$  M3a: M3b setting $a_{4}=0$ |
| M4: Exponential | Growth of BPD criteria modelled as the interplay between a linear increase and an exponential decrease. At a lower age the linear slope is dominating the course, whereas at a higher age the exponential decrease is dominating the course. | M4b: $f\left( t,s \right)=\left( a_{0}+a_{1}\left( t-a_{2}s \right) \right)exp(-a_{3}\left( t-a_{2}s \right))$  M4a: M4b setting $a_{2}=0$ |
| M5: Gompertz | A steady (monotonic) increase to a target value (asymptotic). | M5b: $f\left( t,s \right)=a_{0}\left( 1-a_{1}\exp\left( -a_{2}\left( t-a_{3}s \right) \right) \right)exp(-a_{4}exp(-a_{2}\left( t-a_{3}s \right)$  M5a: M5b setting $a_{3}=0$ |

*Notes:* Each Model Mx, $x\in\{1,2,3,4,5$} has two variations (a: without sex dependency, b: with sex dependency), i.e. model Mxa is nested in Model Mxb (introducing a constraint of the sex effect to zero). The estimated parameters are the fixed effects $a_{i}, i\in\{0,1,2,3,4,5,6,7,8$} and random effects $u_{k}, k\in\{0,1$}. Some parameters can be expressed in terms of the others, as the function f should be continuous with respect to time t. Observed variables are time $t$ (continuous), therapy commencement $t_{0}$ (continuous), sex $s$ (categorical 0:girls, 1:boys).

Below in eFigure 2 are the various aforementioned models using our baseline data. Again, the models in the second row indicate the same model data, allowing for a time shift by sexes.

**eFigure 2:** *Variations of parametrized models to estimate the natural course of BPD symptoms using the baseline data*


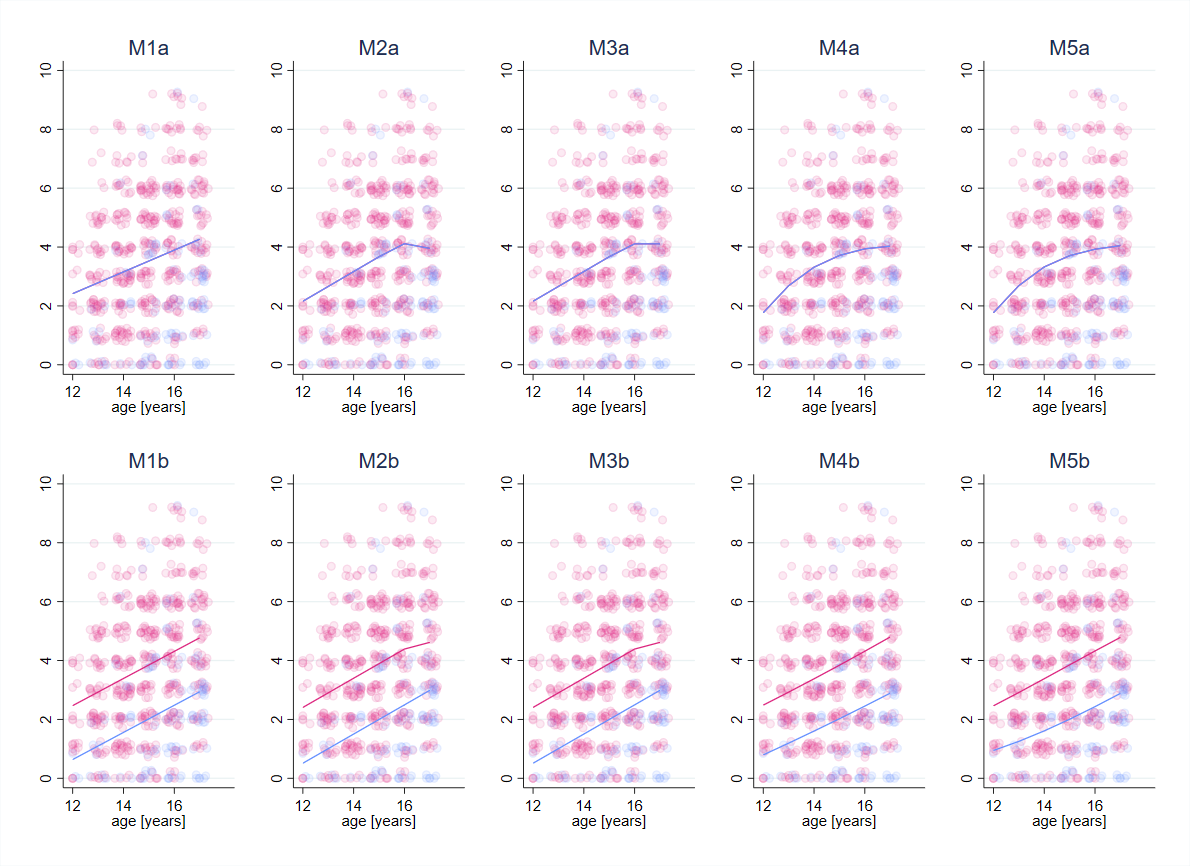


*Notes:* Baseline data points are presented with some jitter. The solid lines represent the fitted model. Y-axis denote the number of fulfilled BPD criteria at baseline. In the bottom row, the model allows for a time shift between girls (red) and boys (blue). Data is modelled by a linear slope (M1), by an increase and later decrease (M2), by an increase until it levels out at some point and decreases again (M3), an exponential decay that interacts with a linear increase (M4), and an increase that levels out (M5).

eTable 2 shows the fit indices to compare models and determine the best fit for the data. Using these fit indices, the best fit for the data is the linear model including a time shift depending on sex (M1b).

**eTable 2:** *Comparative fit indices for the parametrized baseline models*

| Model | AIC | BIC |
| --- | --- | --- |
| M1a: Linear | 2724.3 | 2733.1 |
| M1b: Linear with sex included | 2662.7 | 2676.0 |
| M2a: Breakpoint | 2723.4 | 2741.1 |
| M2b: Breakpoint with sex included | overparametrized | overparametrized |
| M3a: Plateau | overparametrized | overparametrized |
| M3b: Plateau with sex included | overparametrized | overparametrized |
| M4a: Exponential | 2720.8 | 2734.1 |
| M4b: Exponential with sex included | 2664.6 | 2682.3 |
| M5a: Gompertz | 2722.9 | 2740.6 |
| M5b: Gompertz with sex included | overparametrized | overparametrized |

*Notes:* AIC = Akaike Information Criterion; BIC = Bayesian Information Criterion. Overparametrized models refer to the data being insufficient for the complexity of this model.

1. In order to describe the *course of BPD criteria after therapy commencement* (i.e., the therapy effect), we extended the model by additionally including follow-up data. The course of BPD criteria after therapy commencement was modelled by either a linear decrease or an exponential decrease. The decrease itself was allowed to be: age dependent, sex dependent, or therapy dose dependent. It should be noted that dose was included as a sensitivity analysis only (see eText 4 for more detailed information on sensitivity analyses). To account for repeated measures over time, we included random effects: first a random intercept at the natural increase, and second, a random slope for the course of BPD after therapy commencement.

**eFigure 3:** *Variations of parametrized models to estimate the therapy effect using baseline and follow-up data*

*Notes:* Plots show the estimated courses after therapy commencement (y-axis: number of fulfilled BPD criteria) for girls (left column) and boys (right column). The pink and blue lines show the natural courses (i.e., a linear increase) for girls and boys, respectively. The deviation of the natural course (i.e., the therapy effect) is plotted for different times of therapy commencement: 13.6 years (purple), 15 years (orange), and 16.4 years (yellow). Shadowed regions indicate the 95% confidence interval. Data is modelled by a linear age dependent decrease after therapy commencement (M6), a linear age and sex dependent decrease after therapy commencement (M7), an exponential age dependent decrease after therapy commencement (M8), exponential age and sex dependent decrease after therapy commencement (M9), and an exponential age and dose dependent decrease after therapy commencement (M10).


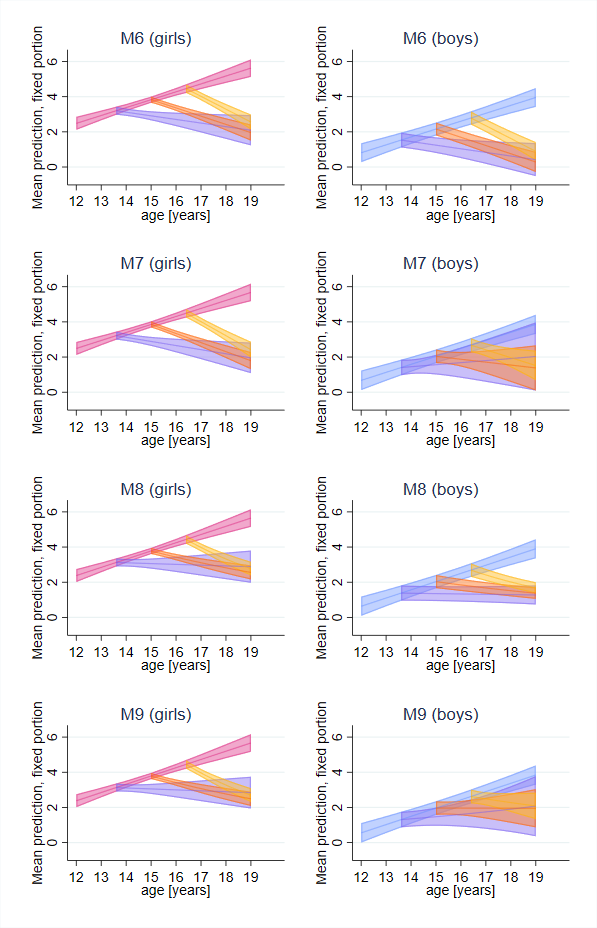


eTable 3 shows the comparative fit indices for each model variation.

**eTable 3:** *Mathematical formulas for description, and model fit indices and for comparison for each model with the inclusion of follow-up data*

| Model | Mathematical formula | AIC | BIC |
| --- | --- | --- | --- |
| M6: Linear age dependent decrease after therapy commencement | $f\left( t,t_{0}, s \right)=\left( a_{0}+u_{0}+a_{1}\left( t-a_{2}s \right) \right)H\left( t_{0}-t \right)+ \left( a_{0}+u_{0}+a_{1}\left( t_{0}-a_{2}s \right) \right)H\left( t-t_{0} \right)+\left( a_{3}+u_{1}+a_{4}(t_{0}-\bar{t}) \right)(t-t_{0})H\left( t-t_{0} \right)$ | 5096.9 | 5137.7 |
| M7: Linear age and sex dependent decrease after therapy commencement | $f\left( t,t_{0}, s \right)=\left( a_{0}+u_{0}+a_{1}\left( t-a_{2}s \right) \right)H\left( t_{0}-t \right)+ \left( a_{0}+u_{0}+a_{1}\left( t_{0}-a_{2}s \right) \right)H\left( t-t_{0} \right)+\left( a_{3}+u_{1}+a_{4}\left( t_{0}-\bar{t} \right)+a_{5}s \right)(t-t_{0})H\left( t-t_{0} \right)$ | 5095.4 | 5141.3 |
| M8: Exponential age dependent decrease after therapy commencement | $f\left( t,t_{0}, s \right)=\left( a_{0}+u_{0}+a_{1}\left( t-a_{2}s \right) \right)H\left( t_{0}-t \right)+ \left( a_{0}+u_{0}+a_{1}\left( t_{0}-a_{2}s \right) \right)H\left( t-t_{0} \right)exp(-\left( a_{3}+u_{1}+a_{4}\left( t_{0}-\bar{t} \right) \right)\left( t-t_{0} \right)))$ | 5080.1 | 5120.8 |
| M9: Exponential age and sex dependent decrease after therapy commencement | $f\left( t,t_{0}, s \right)=\left( a_{0}+u_{0}+a_{1}\left( t-a_{2}s \right) \right)H\left( t_{0}-t \right)+ \left( a_{0}+u_{0}+a_{1}\left( t_{0}-a_{2}s \right) \right)H\left( t-t_{0} \right)exp(-\left( a_{3}+u_{1}+a_{4}\left( t_{0}-\bar{t} \right)+a_{5}s \right)\left( t-t_{0} \right)))$ | 5083.2 | 5129.1 |
| M10: Exponential age and dose (d) dependent decrease after therapy commencement | $f\left( t,t_{0}, d \right)=\left( a_{0}+u_{0}+a_{1}\left( t-a_{2}s \right) \right)H\left( t_{0}-t \right)+ \left( a_{0}+u_{0}+a_{1}\left( t_{0}-a_{2}s \right) \right)H\left( t-t_{0} \right)exp(-\left( a_{3}+u_{1}+a_{4}\left( t_{0}-\bar{t} \right)+a_{5}d \right)\left( t-t_{0} \right)))$ |  |  |

*Notes:* The estimated parameters are the fixed effects $a_{i}, i\in\{0,1,2,3,4,5,6$} and random effects $u_{k}, k\in\{0,1$}. Observed variables are time $t$ (continuous), therapy commencement $t_{0}$ (continuous), sex $s$ (categorical 0: girls, 1: boys), and dose $d$ (inpatient treatment, categorical 0: no, 1: yes). The constant $\bar{t}$ is set to 15 years. Model M10 including dose dependency is only included as part of sensitivity analyses. As the observed variable dose has four missing values, AIC and BIC are not reported (not comparable). See eText 3 for more detailed information.

According to the fit indices shown above, the best model fit was an exponential, age dependent decrease without a sex effect (M8), and was therefore chosen as the final model.

**eText 3: Effect of age on treatment intensity (dose)**

We conducted a sensitivity analysis in order to investigate whether or not the association between age and BPD criteria is affected by treatment dose. Treatment dose was quantified by the number of sessions (outpatient treatment) and number of days in the clinic (inpatient treatment), as assessed at the follow-up timepoints. The sensitivity analysis was done over two steps.

First, we investigated whether age has an effect on treatment dose. This was done by calculating two zero-inflated negative binomial regressions with age, timepoint, and age x timepoint interactions as predictors for the two outcomes: number of sessions for outpatient treatment, and number of days in the clinic for inpatient treatment. When running a zero-inflated negative binomial regression, first a logit model is fitted, followed by the negative binomial model. A logit model was used to estimate the excess of patients without any treatment to what can be expected by the negative binomial model alone. The zero-inflated negative binomial regression revealed no association between age and outpatient treatment dose at either timepoint. In contrast, for inpatient treatment dose, the logit model revealed a significant effect of age at follow-up 1, indicating that younger patients were less likely to receive any inpatient treatment at all during the first year after baseline (OR = 0.83, SE=0.07, CI = [0.71, 0.98], *p*=0.029). However, the negative binomial model revealed no association between age and inpatient treatment dose at either timepoint.

Second, because a significant effect of age on the probability of receiving inpatient treatment during the first year after baseline was found, inpatient treatment (i.e., d: yes(1)/no(0)) was included as an additional predictor in the main analyses. The results remained unchanged by this, indicating that the effect of age on the therapy effect was not significantly affected by inpatient treatment (see Model 10 above: Exponential age and dose dependent decrease after therapy commencement, AIC=5057.2, BIC=5103.1).

**eText 4: Check for systematic loss of participants**

To check for a systematic loss of participants, the subjects with only baseline data were compared to those who have at least one follow-up assessment with respect to several variables including: school type (1 "Hauptschule," 2 "Realschule," 3 "Gymnasium," 4 "other"), living situation (1 "both parents," 2 "mother only," 3 "father only," 4 "other (i.e., step-parents)"), BPD diagnosis (>=5 criteria met), NSSI diagnosis (number of NSSI past year >=5), suicide attempt (within the past 2 years), number of diagnoses, ICD-10 FX diagnosis (yes or no, X = {0,1,2,3,4,5,6,7,8,9}^a^), medication (yes/no). We conducted a stepwise logistic regression minimizing the Bayes Information Criterion. The only significant predictor for being in the group with only baseline data selected by the backward model selection algorithm was the F9 diagnosis (‘Behavioral and emotional disorders with onset usually occurring in childhood and adolescence,’ e.g. attention-deficit hyperactivity disorders, conduct disorders), with an OR of 0.51 (SE = 0.97, CI = [0.36,0.75], *p*<0.001). That is, if someone were to have been diagnosed with an F9 diagnosis, they were more likely to have dropped out of the study (and therefore having only baseline data).

^a^ FX diagnoses 0-9 indicate the following: 0 = mental disorders due to known physiological conditions, e.g. dementia; 1 = substance related disorders, e.g. drugs or alcohol; 2 = psychotic disorders; 3 = affective disorders; 4 = anxiety, dissociative, stress-related, somatoform and other nonpsychotic mental disorders; 5 = behavioural syndromes associated with physiological disturbances and physical factors, e.g. eating disorders, sleep disorders, sexual dysfunction; 6 = personality disorders (other than BPD); 7 = intellectual disabilities; 8 = pervasive and specific developmental disorders, e.g. disorders of speech and language, scholastic skills; 9 = Behavioral and emotional disorders with onset usually occurring in childhood and adolescence, e.g. ADHD, conduct disorder.

**Supplementary Materials references**

Aleva, A., Laceulle, O. M., Denissen, J. J., Hessels, C. J., & van Aken, M. A. (2022). Adolescence as a peak period of borderline personality features? A meta-analytic approach. *European Journal of Personality*, 08902070221134652. https://doi.org/10.1177/08902070221134652

Álvarez-Tomás, I., Ruiz, J., Guilera, G., & Bados, A. (2019). Long-term clinical and functional course of borderline personality disorder: A meta-analysis of prospective studies. *European Psychiatry*, *56*(1), 75–83. https://doi.org/10.1016/j.eurpsy.2018.10.010

Alvarez-Tomás, I., Soler, J., Bados, A., Martín-Blanco, A., Elices, M., Carmona, C., Bauzà, J., & Pascual, J. C. (2017). Long-Term Course of Borderline Personality Disorder: A Prospective 10-Year Follow-Up Study. *Journal of Personality Disorders*, *31*(5), 590–605. https://doi.org/10.1521/pedi_2016_30_269

Arens, E. A., Stopsack, M., Spitzer, C., Appel, K., Dudeck, M., Völzke, H., Grabe, H. J., & Barnow, S. (2013). Borderline Personality Disorder in Four Different Age Groups: A Cross-Sectional Study of Community Residents in Germany. *Journal of Personality Disorders*, *27*(2), 196–207. https://doi.org/10.1521/pedi_2013_27_072

Bornovalova, M. A., Hicks, B. M., Iacono, W. G., & McGue, M. (2009). Stability, Change, and Heritability of Borderline Personality Disorder Traits from Adolescence to Adulthood: A Longitudinal Twin Study. *Development and Psychopathology*, *21*(4), 1335–1353. https://doi.org/10.1017/S0954579409990186

d’Huart, D., Seker, S., Bürgin, D., Birkhölzer, M., Boonmann, C., Schmid, M., Schmeck, K., & Bach, B. (2023). Key insights from studies on the stability of personality disorders in different age groups. *Frontiers in Psychiatry*, *14*. https://www.frontiersin.org/articles/10.3389/fpsyt.2023.1109336

D’Agostino, A., Pepi, R., & Starcevic, V. (2022). Borderline personality disorder and ageing: Myths and realities. *Current Opinion in Psychiatry*, *35*(1), 68–72. https://doi.org/10.1097/YCO.0000000000000764

Gunderson, J. G., Stout, R. L., McGlashan, T. H., Shea, M. T., Morey, L. C., Grilo, C. M., Zanarini, M. C., Yen, S., Markowitz, J. C., Sanislow, C., Ansell, E., Pinto, A., & Skodol, A. E. (2011). Ten-Year Course of Borderline Personality Disorder. *Archives of General Psychiatry*, *68*(8), 827–837. https://doi.org/10.1001/archgenpsychiatry.2011.37

Johnson, J. G., Cohen, P., Kasen, S., Skodol, A. E., Hamagami, F., & Brook, J. S. (2000). Age-related change in personality disorder trait levels between early adolescence and adulthood: A community-based longitudinal investigation. *Acta Psychiatrica Scandinavica*, *102*(4), 265–275. https://doi.org/10.1034/j.1600-0447.2000.102004265.x

Sharp, C., Vanwoerden, S., & Wall, K. (2018). Adolescence as a Sensitive Period for the Development of Personality Disorder. *The Psychiatric Clinics of North America*, *41*(4), 669–683. https://doi.org/10.1016/j.psc.2018.07.004

Soloff, P. H., & Chiappetta, L. (2019). 10-Year Outcome of Suicidal Behavior in Borderline Personality Disorder. *Journal of Personality Disorders*, *33*(1), 82–100. https://doi.org/10.1521/pedi_2018_32_332

Stepp, S. D., Keenan, K., Hipwell, A. E., & Krueger, R. F. (2014). The impact of childhood temperament on the development of borderline personality disorder symptoms over the course of adolescence. *Borderline Personality Disorder and Emotion Dysregulation*, *1*. https://doi.org/10.1186/2051-6673-1-18

Temes, C. M., & Zanarini, M. C. (2018). The Longitudinal Course of Borderline Personality Disorder. *The Psychiatric Clinics of North America*, *41*(4), 685–694. https://doi.org/10.1016/j.psc.2018.07.002

Videler, A. C., Hutsebaut, J., Schulkens, J. E. M., Sobczak, S., & van Alphen, S. P. J. (2019). A Life Span Perspective on Borderline Personality Disorder. *Current Psychiatry Reports*, *21*(7), 51. https://doi.org/10.1007/s11920-019-1040-1

Winsper, C. (2021). Borderline personality disorder: Course and outcomes across the lifespan. *Current Opinion in Psychology*, *37*, 94–97. https://doi.org/10.1016/j.copsyc.2020.09.010

Zanarini, M. C., Frankenburg, F. R., Reich, D. B., & Fitzmaurice, G. (2010). The 10-year Course of Psychosocial Functioning among Patients with Borderline Personality Disorder and Axis II Comparison Subjects. *Acta Psychiatrica Scandinavica*, *122*(2), 10.1111/j.1600-0447.2010.01543.x. https://doi.org/10.1111/j.1600-0447.2010.01543.x

Zanarini, M. C., Temes, C. M., Frankenburg, F. R., Reich, D. B., & Fitzmaurice, G. M. (2018). Description and prediction of time-to-attainment of excellent recovery for borderline patients followed prospectively for 20 years. *Psychiatry Research*, *262*, 40–45. https://doi.org/10.1016/j.psychres.2018.01.034
